# Rheumatoid Arthritis Patient With Neurofibromatosis Type 1: Case Report and Review of the Literature

**DOI:** 10.7759/cureus.51986

**Published:** 2024-01-09

**Authors:** Yan-Jun Li, Shu-Jie Li, Dong-Mei Li, Hong-Xia Yan, Dong-Mei Shi

**Affiliations:** 1 School of Clinical Medicine, Weifang Medical University, Weifang, CHN; 2 Department of Rheumatology and Immunology, Jining No. 1 People's Hospital, Jining, CHN; 3 Department of Microbiology and Immunology, Georgetown University Medical Center, Washington, D.C., USA; 4 Department of Dermatology, Jining No. 1 People's Hospital, Jining, CHN

**Keywords:** neurofibromatosis type 1 (nf1), vitamin d, osteoporosis (op), rheumatoid arthritis, coronary heart disease (chd)

## Abstract

A 66-year-old neurofibromatosis type 1 (NF1) patient with polyarticular pain for nine years, aggravated for two days, was transferred from the Emergency Intensive Care Unit (EICU) to our rheumatology department. She was diagnosed with NF1 nine years ago by a gene mutation detection and coronary heart disease (CHD) three months ago. The patient was diagnosed with rheumatoid arthritis (RA) this time. After 24 days of treatment with appropriate medication, the patient was discharged with relieved joint pain. However, about four months later, the patient died of circulatory failure caused by myocardial infarction. We analyzed the possible reasons for her outcome and made a review of the literature. There are few clinical reports of NF1 complicated with RA. We found five cases reported in the literature up to date during our search and included them in our communication to compare with our case. NF1 combined with RA mainly affects adult women and usually starts with NF1 and is followed by RA after at least six years of NF1 symptom onset. Although the summarized characteristics of clinical and potential pathogenesis of NF1 combined with RA were limited with these six cases, we hope that this will help clinicians to increase their understanding of this rare complication, thus helping to guide clinical medication.

## Introduction

Neurofibromatosis (NF) is a group of heterogeneous diseases characterized by damage to the nervous system, skin, mouth, eyes, and bones, which mainly include NF1 (type 1), NF2 (type 2), and schwannomatosis [1]. Among them, NF1 is the most common type. Rheumatoid arthritis (RA) is a common chronic and systemic autoimmune disease characterized by cartilage and bone destruction. Since both diseases have distinct pathogenesis and clinical features, it is not common for medical professionals to connect them. Here, we report an adult female who initially developed NF1 symptoms at about 16 years old and was diagnosed with RA after 46 years. Moreover, a review the literature on NF1 associated with RA is presented.

## Case presentation

**Figure 1:**
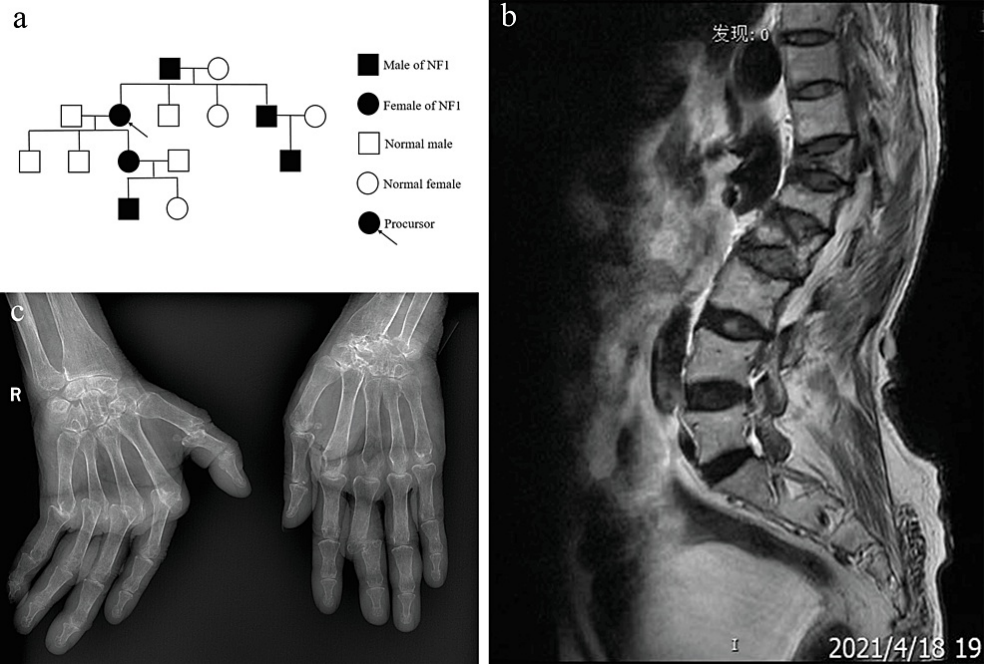
Family tree of the patient and imaging features Family trees of the patient (a). Thoracolumbar kyphosis, with an irregular sequence; edges of vertebral bodies got sharp, and signals in vertebral bodies became uneven; wedge-shaped changes in different degrees of T12-L2 vertebral bodies, and long T1 and short T2 signals in T12 vertebral bodies (b). The bone density of both hands and wrists decreased; the left radiocarpal joint space disappeared; the metacarpophalangeal joint and interphalangeal joint space of both hands narrowed, and the joint surface hardened and rough; dislocation of the metacarpophalangeal joint of the right hand and proximal interphalangeal joint of the left middle finger (c).

A 66-year-old woman with RA was transferred to our rheumatology department from the Emergency Intensive Care Unit (EICU) with complaints of multi-joint pain. This is the third time she has been admitted to our unit. She has had NF1 symptoms for over 50 years, which was confirmed by the clinical presentation and confirmation of gene mutation at (C.1466a > G (Tyr489cys)) seven years ago. Her parents are not close relatives, and many people in her family have a history of NF1 (Figure 1a) but no RA, systemic lupus erythematosus, or psoriasis.

Nine years ago, the patient developed lower back pain with morning stiffness. She visited several hospitals and was diagnosed with lumbar disc herniation. Later, the symptoms progressed to chronic bilateral symmetrical polyarthralgia of large joints, such as the knees, elbows, and ankles. Her activities have been affected when the pain was aggravated. Meanwhile, she presented with morning stiffness of both hands and dry mouth. On June 10, 2015 (six years ago), she visited our unit for the first time. She denied a history of Raynaud’s phenomenon, dry eyes, and rash. Laboratory investigations revealed an elevated C-reactive protein (CRP; 45.40 mg/L) and erythrocyte sedimentation rate (ESR; 40 mm/h). Anti-nuclear antibodies, antibody spectrum, anti-cyclic citrullinated peptide, rheumatoid factor, urine routine test, and HLA-B27 were all negative. Computed tomography showed interstitial changes in both lungs and sacroiliitis. Bone scan revealed polyarticular hypermetabolic inflammatory lesions. Echocardiography demonstrated small amounts of aortic and tricuspid regurgitation and reduced left ventricular diastolic function. She was diagnosed with axial spondyloarthritis according to the 1991 European Spondylarthropathy Study Group (ESSG) criteria for spondylarthropathy. Afterward, several joints on her hands and feet gradually became deformities. The disease had already limited her activities of daily living. At the same time, the patient presented poor appetite, which may be related to her long-term irregular oral administration of prednisone, non-steroidal anti-inflammatory drugs (NSAIDs), and other medications.

In 2019, she received bone cement surgery due to a fracture on the lumbar spine caused by osteoporosis (Figure 1b). On April 14, 2021, the female was hospitalized in our department for the second time complaining of generalized pain for over one month. On physical examination, in addition to the previous facet joint deformity and multi-joint tenderness, the patient's elbow extension was limited, and the muscle strength of the limbs was grade 4. The laboratory evaluation revealed elevated inflammatory markers (CRP 27 mg/L and ESR 37 mm/h). X-rays of the wrists and hands were typical for RA (Figure 1c). She was eventually diagnosed with RA according to the 2010 American College of Rheumatology (ACR)/European League Against Rheumatism (EULAR) criteria. During hospitalization, the patient suffered from recurring severe pain in the upper abdomen, and symptomatic treatment with various medications did not have a good effect. There were no obvious abnormalities in the electrocardiogram and upper abdominal color Doppler ultrasound. Echocardiography showed that the left ventricle ejection fraction was 50%. To perform the gastroscopy, she underwent a coronary computed tomography angiography (CTA) to rule out anesthetic risk, which demonstrated coronary heart disease (CHD), unstable angina, and coronary myocardial bridge. Further coronary angiography showed severe stenosis in multiple coronary branches, and thus gastroscopy examination was not appropriate. Cardiologists made the diagnosis of CHD and suggested coronary artery bypass for her risk of myocardial infarction or even sudden cardiac death. However, the subsequent anticoagulation therapy may aggravate her gastrointestinal symptoms. Finally, the patient refused it and was discharged. After that, she was admitted to the Department of Cardiology of our hospital twice. Her brain natriuretic peptide (BNP) was 323 pg/ml and has been conservatively treated with drugs. During hospitalization, she developed hyponatremia, hypochloremia, and hypocalcemia, which improved after symptomatic treatment.

On June 28, 2021, the patient was admitted to the emergency clinic because of severe vomiting after taking 200 tablets of sulfasalazine and prednisone orally. Electrocardiography revealed a first-degree atrioventricular block. Arterial blood gas analysis was as follows: pH 7.52, partial pressure of carbon dioxide (pCO_2_) 24 mmHg, bicarbonate 19.6 mmo1/L, base excess -3.3 mmo1/L, blood potassium 2.8 mmo1/L, blood sodium 109 mmo1/L, blood clozapine 83 mmol/L, D-dimer 1928.94 ng/ml, and BNP 762 pg/ml. After gastric lavage, she was transferred to EICU for hemoperfusion and symptomatic support treatment, such as correcting the alkalosis.

**Figure 2:**
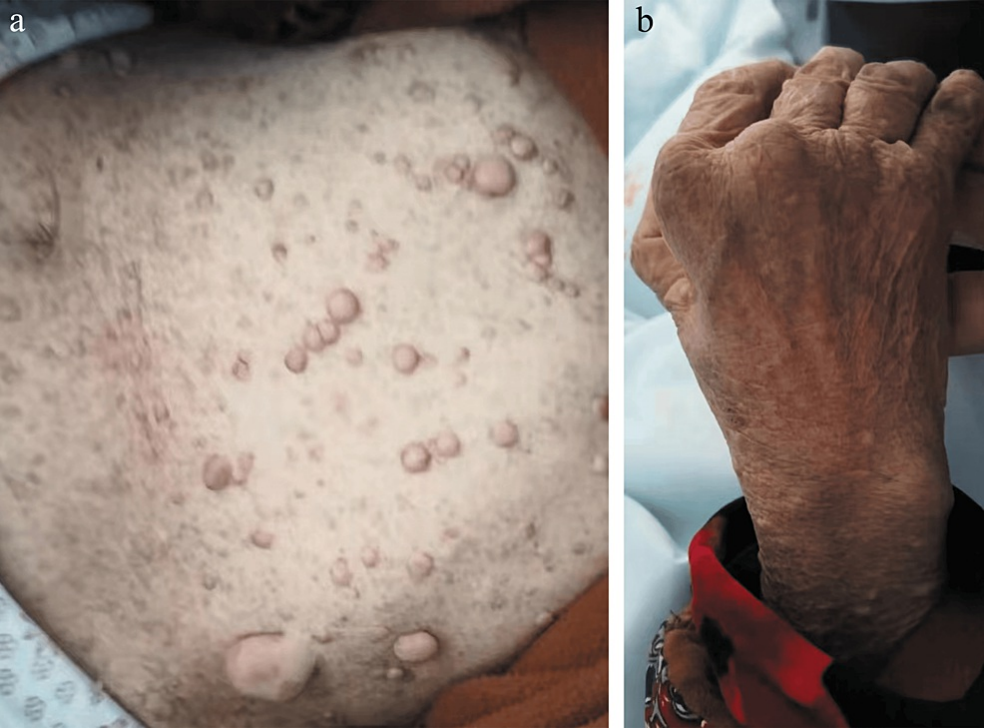
Clinical appearance of the skin lesions and joints The patient was riddled with brown cafe-au-lait spots, patches, and soft nodules of different sizes (a). Ulnar deviation of the right hand; boutonniere deformity of one to five fingers (b).

Three days later, the female was out of a life-threatening condition and complained of increased joint pain. For this reason, she was transferred to our rheumatology department on the same day (July 1, 2021), which was the third time she had been admitted there. Before her transfer, her D-dimer was 1073.48 ng/ml, BNP was 321 pg/ml, blood potassium was 3.19 mmo1/L, and blood sodium was 125 mmo1/L. A physical examination revealed the temperature was 99.9°F, the heart rate was 112 bmp, and the pain score was 5. There were many café-au-lait spots all over the body, ranging from 3 to 15 mm in diameter, with clear boundaries. Hundreds of spherical or semispherical flesh-colored nodules measuring approximately 5-30 mm in diameter can be seen all over the body. They were soft and had no tenderness when touched (Figure 2a). Specialized physical examination showed that she had slight kyphosis on the spine, tenderness of the lumbar spine, multi-joint deformity on the hands and feet, ulnar deviation of the right hand, and boutonniere deformity of one to five fingers (Figure 2b). Her elbow extension was limited, and she complained of tenderness on elbows, shoulders, knees, and ankles when pressed, but there was no noticeable swelling. Muscle strength of limbs was at grade 4. The patient was diagnosed with RA, NF1, and CHD. During hospitalization, the patient has been treated with methylprednisolone 4 mg twice daily, technetium-methylene diphosphate 11 mg/day, elamod 25 mg twice daily, and bulleyaconitine A 0.4 mg three times per day for RA. At the same time, we continued to correct the patient's refractory electrolyte disorder but did not achieve a satisfactory efficacy. When she was discharged from our hospital, her blood sodium was 125 mmo1/L and blood clozapine was 95.1 mmo1/L.

In a three-month follow-up, we learned that three months after her discharge (September 16, 2021), the woman was treated in the Department of Cardiology of our hospital with a complaint of a sustaining burning sensation of the posterior sternum. The electrocardiogram showed extensive anterior myocardial infarction. Laboratory values were as follows: troponin I 4.89 ng/ml, creatine kinase MB fraction (CK-MB) 22.7 ng/ml, myoglobin 98.7 ng/ml, D-dimer 1.26 mg/L, N-terminal pro-BNP＞35000 pg/ml. Hence, emergency physicians quickly performed coronary angiography, indicating that the proximal and middle of the left anterior descending branch was completely occluded. Two stents were implanted afterward for the correction. On September 27, 2021, the patient’s CK significantly increased to 2,565 U/L. One day later, her alanine transaminase (ALT) elevated to 1006.4 U/L and aspartate transaminase (AST) rose to 2555.2 U/L. On September 30, 2021, she suddenly displayed circulatory failure with a heart rate of 35 b/m. Even the vasopressor could not maintain normal blood pressure. Ultimately, the patient's family asked for an automatic discharge after consultation. The patient died when we followed up on October 24, 2021.

## Discussion

The literature search was performed in the databases of PubMed and Embase under the MeSH (Medical Subject Headings) terms/Emtree (descriptor): "neurofibromatosis 1," "arthritis, rheumatoid," and corresponding entry terms/synonyms (entry terms). The MeSH terms and their related entry terms were connected by "OR" to get two search strategies. Then, we joined the two parts with "AND." With the same strategy, we also searched for the equivalent literature in Chinese among SinoMed, CNKI, Wanfang date, and VIP databases to search the Chinese and English literature before December 2022.

There are six cases with NF1 and RA reported in the literature up to date, including the case we reported here (Table 1). All reported cases were women whose onset ages of RA ranged from 20 to 62, with an average of about 35. That is probably because RA is mainly involved in adult women. Five patients developed NF1 symptoms before RA. Among them, the longest interval time was 46 years, and the shortest was six years, with a median of 14 years. One patient was made the diagnosis of RA before developing multiple, non-tender, flesh-colored nodules that were arranged in a dermatomeric distribution, which eventually diagnosed with segmental neurofibromatosis (SNF). Her eruption of SNF was induced by biological agents; however, there was no progress after drug withdrawal.

**Table 1:** Clinical characteristics of six cases of NF complicated with RA NF, neurofibromatosis; RA, rheumatoid arthritis; Y, years old; F, female; NF1, neurofibromatosis type 1

Number	Study	Gender	Age (Y)	Diseases that show symptoms first	The onset age of NF symptoms (Y)	The onset age of RA (Y)	Interval time of onset (Y)
1	Fan, 1996 [2]	F	65	NF	35	42	7
2	Fan, 1996 [2]	F	39	NF	17	31	14
3	Fan, 1996 [2]	F	25	NF	14	20	6
4	Drago et al., 2016 [3]	F	78	RA	78	-	-
5	Rischin et al., 2019 [4]	F	45	NF1	5	20	15
6	Present case	F	66	NF1	About 16	62	About 46

Three patients were familial cases reported by Fan Jianchun [2] in 1996. The mother and her two daughters suffered from multiple neurofibroma. Multiple neurofibroma is the old name of NF. Unfortunately, the specific classification was not described in this paper. Still, we speculate that NF1 is the most likely diagnosis according to the incidence of NF1 and their multiple café-au-lait spots and brown macules. They were diagnosed with RA within 15 years after the clinical manifestations of NF, and all three developed secondary autoimmune hemolytic anemia.

In 2016, Drago et al. [3] reported one case who presented with segmental NF symptoms after a six-month therapy with intravenous infliximab for RA. Multiple painless flesh-colored nodules appeared in the upper right torso and right arm. After the withdrawal of this drug, no new lesions were appearing. The authors thought that infliximab may play a role in the early stage of NF1.

The fifth case was reported by Rischin et al. [4] in 2019. The patient was diagnosed with NF1 at age five and then developed RA. About six weeks after starting tofacitinib, the patients experienced extensive skin fibroma eruption, gradually subsiding after several months of drug withdrawal.

NF1 is an autosomal dominant disorder. Reports of NF1 with RA were still relatively rare, although there were some cases of NF1 complicated with autoimmune diseases, such as multiple sclerosis, IgA nephropathy, autoimmune hemolytic anemia, bullous pemphigoid, vitiligo, and alopecia areata [5]. The NF1 gene is located in human chromosome 17 [6]. This gene encodes neurofibromin that can promote Ras inactivation [1]. On the contrary, NF1 gene defect will lead to the over-activation of Ras, thus activating the downstream MAPK pathway and PI3K/Akt/mTOR pathway and promoting cell proliferation and growth [1].

The rash of the fourth and fifth patients is related to the treatment of RA. Cutaneous neurofibromas erupted around the body of the fifth patient after the six-week therapy with tofacitinib and then gradually regressed when she stopped the drug. As a Janus kinase (JAK) inhibitor, tofacitinib effectively suppresses inflammation by inhibiting Janus kinase-signal transducers and activators of transcription (JAK-STAT) pathway from decreasing the production of relative cytokines [7]. Due to this pathway involved in the pathogenesis of RA, tofacitinib was approved in many countries and was widely used for treating RA. However, the authors mentioned that tofacitinib can affect the Ras pathway through a process known as intracellular crosstalk, which may lead to transient neurofibromas [4]. This potential possibility reminds clinicians that using JAK and TNF-α inhibitors may induce or aggravate the neurofibromas of NF1. The above drugs should be used with caution if the RA patients have a personal or family history of NF1. Of course, more evidence is needed to prove this hypothesis.

As we all know, RA often leads to bone destruction. Various inflammatory factors and cells, including B cells, IL-17, and TNF-α, will cause an imbalance between osteoblasts and osteoclasts, thus causing bone destruction [8,9]. Meanwhile, experiments show that compared with healthy controls, RA patients have a decrease in the concentration of 25-(OH) vitamin D in peripheral blood, and the degree of decline is related to disease severity [10]. Many studies have shown that the serum 25-(OH) vitamin D in NF1 patients is significantly lower than that of healthy controls [11,12], which may lead to the occurrence of bone lesions in NF1 patients by the reduction of bone mineral density.

Therefore, we are convinced that both NF1 and RA can lead to bone destruction and bone mass decline through various mechanisms. When the two diseases occur together, there may be a synergistic effect on bone damage, manifesting as more severe osteoporosis and a higher fracture incidence. It may be rational for clinicians to use anti-osteoporosis drugs (such as vitamin D and its active products, calcium, bisphosphonate, and calcitonin) when treating such patients, especially those who had been treated with glucocorticoid for ameliorating symptoms of RA. These anti-osteoporosis drugs may reduce fracture incidence and improve the patient's quality of life. Meanwhile, patients should be encouraged to participate in outdoor activities and supplement vitamin D through sunlight exposure.

Our patient was found to develop coronary heart disease many years later of NF1 and RA, and three-month follow-ups found that she died due to circulatory failure caused by acute myocardial infarction. The correlation between NF1 and coronary heart disease is poorly studied. Some literature showed that patients with NF1 are more prone to cardiovascular abnormalities than healthy people, manifesting as stenosis, spontaneous dissection, vasospasm, thrombosis, or aneurysm [13]. Those abnormalities usually involve the middle and large arteries, leading to pulmonary valve stenosis, aortic coarctation, ventricular septal defect, eccentric hypertrophy of the left ventricle, and intracardiac tumor [14]. However, the patient's imaging examination showed no such abnormality.

There are still more than a dozen case reports describing coronary aneurysms related to NF1, which can lead to myocardial infarction or sudden cardiac death in some cases [13]. Three months before her death, a coronary angiography examination showed severe stenoses in multiple coronary branches and a muscular bridge without evidence of a coronary aneurysm.

**Figure 3:**
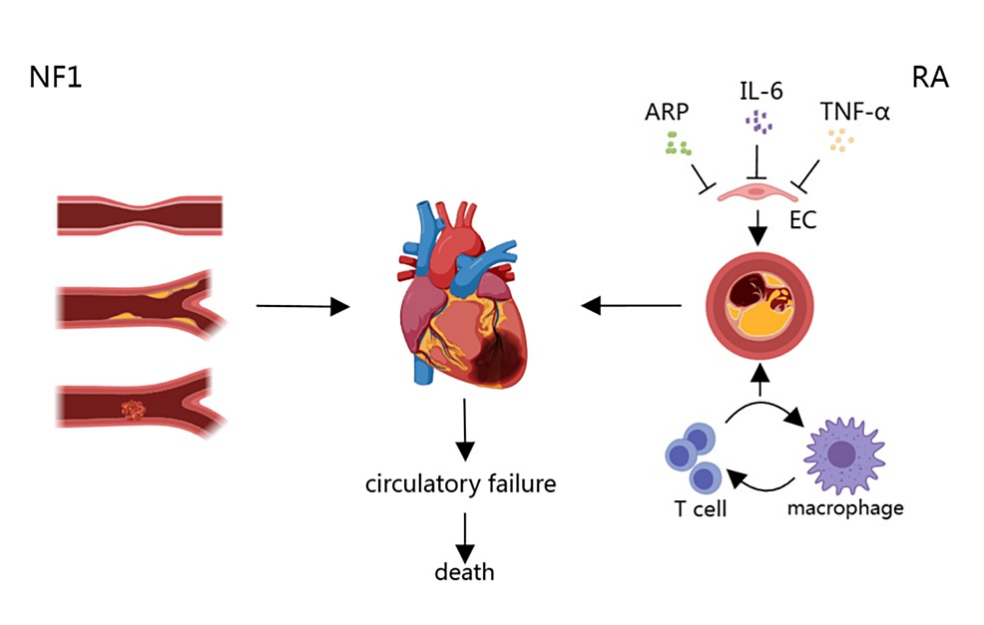
Possible mechanism of acute cardiovascular events caused by NF1 complicated with RA Image credits: Yan-Jun Li NF1 often involves vasospasm, stenosis, and thrombosis. As for RA, the increased IL-6, TNF-α, and APR in serum will promote endothelial activation, making atherosclerotic plaques unstable. Moreover, the interaction between T cells and macrophages can also increase plaque instability. Together, these factors enable the occurrence of acute myocardial infarction, leading to circulatory failure and even death. NF1, neurofibromatosis type 1; RA, rheumatoid arthritis; APR, acute phase reactants; IL-6, Interleukin- 6; TNF-α, tumor necrosis factor-α; EC, endothelial cells

Unsurprisingly, the risk of cardiovascular disease in patients with RA is more common than NF1. It is 1.5 to two times higher in RA patients than healthy controls with similar demographic characteristics [15]. Compared with the general population, the risk of myocardial infarction in RA patients increased by 68% [16], and the risk of death from cardiovascular diseases increased by 50% [17]. The reason may be associated with chronic and systemic inflammation of RA or long-term drug usage. For example, IL-6, TNF-α, and acute phase reactants in the circulating inflammatory pathway of RA patients increase endothelial activation, which may lead to plaque instability [18]. Another explanation is the interaction between T cells and macrophages, which play a critical role in the pathogenesis of RA, which can also cause the instability or even rupture of atherosclerotic plaques, leading to acute coronary syndrome [19]. Researchers speculated that the inflammation of atherosclerotic plaques may have the same mechanism as RA [19] (Figure 3).

To sum up, combined with the patient's medical history, we speculate that the outcome of our patient may be related to the history of RA, long-term steroid treatment, and irreversible damage of vital organs due to the toxic effects of drug overdose.

## Conclusions

This paper reports a rare case of NF1 with RA and reviews five other similar cases. We analyze the characteristics of each case and the death of our patient to increase clinicians’, especially rheumatologists’, awareness of the treatment options and possible complications of this infrequent comorbidity. Again, we emphasize that rheumatologists should give the potential side effects ongoing attention.
